# Comparative Risk Assessment of the Burden of Disease from Climate Change

**DOI:** 10.1289/ehp.8432

**Published:** 2006-07-11

**Authors:** Diarmid Campbell-Lendrum, Rosalie Woodruff

**Affiliations:** 1 Department of Public Health and Environment, World Health Organization, Geneva, Switzerland; 2 London School of Hygiene and Tropical Medicine, London, United Kingdom; 3 National Centre for Epidemiology and Population Health, Australian National University, Canberra, Australia

**Keywords:** burden of disease, climate change, national, quantitative comparative risk assessment, regional

## Abstract

The World Health Organization has developed standardized comparative risk assessment methods for estimating aggregate disease burdens attributable to different risk factors. These have been applied to existing and new models for a range of climate-sensitive diseases in order to estimate the effect of global climate change on current disease burdens and likely proportional changes in the future. The comparative risk assessment approach has been used to assess the health consequences of climate change worldwide, to inform decisions on mitigating greenhouse gas emissions, and in a regional assessment of the Oceania region in the Pacific Ocean to provide more location-specific information relevant to local mitigation and adaptation decisions. The approach places climate change within the same criteria for epidemiologic assessment as other health risks and accounts for the size of the burden of climate-sensitive diseases rather than just proportional change, which highlights the importance of small proportional changes in diseases such as diarrhea and malnutrition that cause a large burden. These exercises help clarify important knowledge gaps such as a relatively poor understanding of the role of nonclimatic factors (socioeconomic and other) that may modify future climatic influences and a lack of empiric evidence and methods for quantifying more complex climate–health relationships, which consequently are often excluded from consideration. These exercises highlight the need for risk assessment frameworks that make the best use of traditional epidemiologic methods and that also fully consider the specific characteristics of climate change. These include the long-term and uncertain nature of the exposure and the effects on multiple physical and biotic systems that have the potential for diverse and widespread effects, including high-impact events.

The process of climate change, including both increases in global average temperatures (“global warming”) and changes in other climate characteristics such as the spatial and temporal distribution of precipitation, has important implications for human health. It is important to describe, measure, and predict the health effects of climate change for two reasons. First, this provides a fuller picture of the consequences of mitigating, or failing to mitigate, emissions of greenhouse gases that are the main anthropogenic contribution to climate change. The long persistence of these gases in the atmosphere means that current mitigation activities (or lack of them) will have consequences for all natural and human systems over coming decades and centuries. They should ideally be informed by measures of the overall size and global distribution of likely health effects of climate change throughout suitably long periods to be considered alongside other impacts such as on biodiversity (Parmesan and Yohe 2003; Thomas et al. 2004a). Even imperfect estimates of the full range of global impacts can provide useful information, provided they are accompanied by clear descriptions of the associated assumptions and uncertainties. Second, quantitative studies can help inform policies to adapt to climate changes that are now inevitable because of both natural variability and past greenhouse gas emissions. Such actions typically affect the national or subnational level and require information on the likelihood and expected magnitude of specific health impacts in the local context, allowing for the more appropriate allocation of resources to prevent harm from effects such as extreme weather-related events and changes in disease distributions.

Recent comparisons of natural and anthropogenic influences on regional climate (Stott et al. 2004) have demonstrated that human activity increased the probability of a specific past climate event, with severe health consequences (> 44,000 deaths in the European heat wave of summer 2003) (Kosatsky 2005). However, estimating the full range of effects of climate change on health over long time scales presents additional challenges to epidemiologic methods. These include the absence of an appropriate comparison group, the long period over which human actions affect the climate, the large number of health outcomes potentially affected by climatic change, and the numerous nonclimatic influences on each of these outcomes. For these reasons it is misleading simply to observe long-term trends in climate-related diseases and to attribute these changes directly to anthropogenic climate change (e.g., Kovats et al. 2001; McMichael and Githeko 2001; Reiter 2001). The most plausible estimates of future climate change impacts are instead based on empirically observed relationships between weather or climate conditions and health effects, either in space and/or in time, or, for infectious diseases, on models that capture a detailed understanding of the effects of climate on the biologic processes that determine disease transmission (Rogers and Randolph 2000; Small et al. 2003). Projections of global climate models can be linked to these relationships to indicate how future climate change may influence the level of health outcomes—such as changes in the population living in areas with climates suitable for the transmission of malaria parasites or dengue virus (e.g., Hales et al. 2002; Martens 1998; Rogers and Randolph 2000) or the numbers of people exposed to coastal flooding (Nicholls et al. 1999). These models already provide useful quantitative measures of future risk. However, the results of these models are difficult to relate directly to inform decisions on mitigation (e.g., greenhouse gas emission reduction strategies) because *a*) many do not attempt to account for changes in nonclimatic influences such as economic development (and hence the ability to protect against disease risk), and *b*) the model outcomes are often indirectly related to health, and then only to specific diseases. It is therefore difficult to judge the overall magnitude of the likely health impacts of climate change, either globally or in a specific country (e.g., the combined health effect of a projected 10% increase of the population exposed to coastal flooding, a 20% increase in population living in areas suitable for dengue transmission, and a 5% drop in deaths in cold extremes) and compare these with other threats.

These concerns can be addressed partly by using a standard framework for comparison across risk factors and diseases. The World Health Organization (WHO) has recently developed an approach for comparative risk assessment that has been applied to estimate the current and future disease burden from 25 diverse risk factors, including climate change, in a comparable and transparent manner (Ezzati et al. 2002, 2004; Murray and Lopez 1997; WHO 2002). The assessment generated estimates of the numbers of deaths and disability-adjusted life years (DALYs) attributable to each risk factor in the year 2000, along with expected changes in exposures and associated relative risks of disease outcomes, for several time points between 2000 and 2030. A similar approach has been applied to estimate the effects of climate change on health within the Oceania region in the Pacific Ocean for 2020 and 2050. This assessment principally focused on the impacts on Australian populations, although quantitative estimates were generated for a subset of the health outcomes for New Zealand and Pacific Island countries.

This article outlines the comparative risk assessment approach and discusses its main advantages and limitations for its use in assessments of the health impacts of climate change at the national level. The comparative risk assessment method involves four stages: *a*) identifying health outcomes sensitive to climatic influences, *b*) quantifying the dose–response relationship for a baseline climate period, *c*) defining future exposure scenarios, and *d*) estimating the burden of disease that is attributable to a risk factor (i.e., relative to the risk if climate were unaffected by human actions) and the burden that is avoidable by plausible reductions in the risk factor. Each of these steps requires more detailed decisions, for example, selection between various possible scenarios for future greenhouse gas emissions and associated climate change, or between alternative models describing the relationships between climate and individual health outcomes. These are described only briefly here; more detail on the specific methods used in both the global and the regional assessment are reported by McMichael et al. (2003b, 2004).

## Identifying Climate-Sensitive Health Outcomes

Time-series studies and geographic comparisons provide good evidence that a range of health impacts are sensitive to variations in meteorologic conditions of a scale comparable to the climate changes that are expected over the coming century or so (i.e., a 1.4–5.8°C increase in global mean temperatures, changes in regional patterns of rainfall, and potential increases in the frequency of severe storms [Intergovernmental Panel on Climate Change (IPCC) 2001a; Knutson and Tuleya 2004]. These impacts include deaths and morbidity associated with weather extremes such as heat waves, cold waves, and floods, the incidence of all infectious diseases transmitted by insects and other invertebrate vectors or caused by pathogens that replicate in food or water, and the effects of malnutrition, particularly in poor populations that rely on subsistence farming (McMichael et al. 2003a). These therefore provide an initial list of climate-sensitive health outcomes that should be considered in comparative risk assessment exercise. It should be noted that taking this disease-by-disease approach already tends toward conservative estimates of the full range of health effects. Climate can influence an even wider range of diseases through multiple pathways (e.g., Gommes et al. 2004), and climate is such a major influence on all ecosystem functions that climate change, and particularly sudden “threshold” shifts, may lead to the emergence of new disease threats that are not currently foreseeable.

## Quantitative Estimation of Climate–Health Relationships

Comparative risk assessment requires quantitative models of the climatic effects for health outcomes (relevant to the study population) or sufficient reliable disease and environmental data to allow their construction. These models are usually generated based on measurements of the health effects of observed variations in climate in time [e.g., the effect of unusually hot or cold days on disease rates (Checkley et al. 2000; Hajat et al. 2005)] or space (Hales et al. 2002; Rogers and Randolph 2000), or both (e.g., Kuhn et al. 2003; Singh et al. 2001). The extrapolation of short-term or geographic relationships between climate and disease to the process of long-term climate change is probably one of the most important sources of uncertainty in the process because impacts from more gradual processes may be either less severe (e.g., because of gradual adaptation) or more severe (e.g., because of long-term stress leading to irreversible changes in food-producing ecosystems) than expected. Also, some projected climatic conditions such as heat waves are of a duration or intensity not previously experienced by many (researched) human populations, which presents challenges to the estimation of future risk.

If the assessment seeks to make direct comparisons with disease burdens attributable to other risk factors (as was the case in the global assessment), then it is usually necessary to use a summary measure of population health such as the DALY (Murray 1994) to combine effects of both mortality and morbidity from the various health impacts. This further restricts assessment to diseases with well-characterized and quantified disease burdens (e.g., cases of diarrhea), excluding many likely outcomes of climate change that are relevant to health (e.g., populations suffering increased water stress; Arnell 1999) but that do not yet have well-defined relationships to disease risk. Where different models exist for the same health outcome, final selection should be made on the basis of *a*) validation against historical data, *b*) plausibility of biological assumptions, and *c*) plausibility of extrapolation to other regions. The outcomes considered in the global and regional assessment are shown in Table 1.

## Defining Exposure Scenarios

The global and regional analyses used climate models to define alternative plausible distributions of the risk factor in geographic regions over several decades. Many risk factors for health can potentially be altered over relatively short periods (i.e., up to several years). In these cases the focus is usually on calculating current attributable and avoidable burdens to indicate the benefits of addressing the risk factor in the near future. In contrast, decisions on greenhouse gas emissions influence global climate over many decades, and the impacts on human societies are therefore likely to be increasingly evident over similar periods. In this case the full implications of policy change are made clear only when exposure and associated effects are considered over the medium to long term.

A logical baseline “exposure” for comparison would consist of a climate not yet affected by any human activities. This is commonly approximated by using the previous World Meteorological Organization–approved standard period from 1961 to 1990 as the baseline (World Meteorological Organization 1989). The IPCC has concluded that climate changes since around the middle of this period (i.e., 1975) are at least partly attributable to human action (IPCC 2001b). Therefore, this baseline tends to produce conservative estimates of attributable future risk.

Exposure scenarios are based on global climate scenarios: internally consistent representations of future climatic conditions. These are generated by applying a range of levels of anthropogenic “forcings” (most important, greenhouse gas emissions) to computer models representing human and natural influences on the global climate. The output data consist of grid maps of variables, such as temperature, precipitation, and humidity, at a greater or lesser spatial resolution. The global assessment, for example, applied three “scenarios” of future greenhouse gas emission levels (Arnell et al. 2002): *a*) continuing on an unmitigated trajectory approximately following the IPCC 1S92a scenario in which effective atmospheric carbon dioxide (CO_2_) concentration rises at 1% per year after 1990 (Arnell et al. 2002); *b*) stabilization of CO_2_ concentrations at 750 ppm (approximately double preindustrial concentrations; Carbon Dioxide Information Analysis Center 2003) by 2210 (scenario s750); or *c*) stabilization of CO_2_ concentrations at 550 ppm by 2170 (scenario s550), with projected changes in climate variables overlaid on a grid of 1961–1990 climate condition at 0.5° spatial resolution.

The Oceania regional assessment also used preexisting climate scenarios generated by global climate models [this time with updated emissions scenarios from the *Special Report on Emission Scenarios* (SRES)] (Nakicenovic et al. 2000; see also “Appendix”). The Oceania assessment used “down-scaled” global climate model patterns to generate country-level projections of future changes in temperature and rainfall. All global climate models show a general warming trend in the Australian region. To capture the wide variation in rainfall patterns across the continent estimated by different models, the regional assessment used two models, CSIROMk2 and ECHAM4, that represent the spectrum of different precipitation projections. The geographic resolution of the output was 0.25° (~ 25 km^2^), a scale fine enough to consider the variation in effects between cities and ecologic zones. This is an important factor in national assessments because budget allocations typically are organized around subnational administrative boundaries.

## Estimating Attributable and Avoidable Burdens of Disease

The comparative risk assessment approach further requires the exposure measurement to be linked to a quantitative climate–health relationship (e.g., the change in disease rates per unit change in the climatic variable), for example, the increase in diarrhea incidence in a country (or subpopulation) per year for each degree centigrade increase in average ambient temperature. This enables the calculation of a relative risk (i.e., proportional change) for the health outcomes under each of the various future climate scenarios. The disease burden attributable to climate change is then estimated by multiplying this relative risk by the total burden of disease that would have been expected to occur in the absence of climate change.

**Figure 1:**
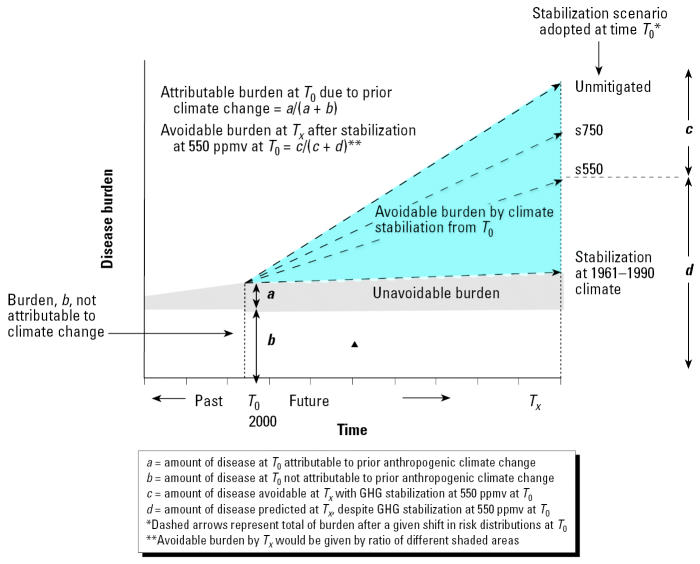
Comparative risk assessment definitions of attributable and avoidable disease burden, in the context of climate change. GHG, greenhouse gases; ppmv, parts per million by volume; *T*, time. Adapted from Kay et al. (2000).

To make inferences about current and future disease burdens, it is also necessary to account for the current and future influences of nonclimatic factors such as socioeconomic development. Nonclimatic effects can be partly addressed by calculating relative risk estimates separately for populations with clearly different baseline disease burdens and vulnerabilities, for example, the 14 WHO subregions in the global assessment or the specific cities and sub-populations considered in the Oceania assessment. Where possible, future relative risks should be applied to projections of disease burden that also account for changes in non-climatic influences over time, such as expected decreases in diarrhea rates as water and sanitation services, ideally, become more widespread in the future (e.g., Murray and Lopez 1997). Finally, changing socioeconomic conditions and physiologic and behavioral adaptations will also affect the vulnerability of populations to the effects of climate change (McMichael and Githeko 2001; Woodward et al. 1998), that is, the relative risk as well as the baseline rate. Both global and national assessments made such adjustments to relative risks of the various outcomes, for example, taking into account projected increases in the proportion of the population that is elderly and therefore particularly susceptible to extreme temperatures, in Oceania, and projected improvements in water and sanitation infrastructure in poor populations, decreasing the climate sensitivity of diarrheal disease, in the global assessment. The concepts of avoidable and attributable disease burdens under alternative climate change scenarios are illustrated graphically in Figure 1.

## Disease-Specific Methods

Detailed descriptions of the methods for quantitative estimation of each of the selected health impacts are reported by McMichael et al. (2003b, 2004). The methodologic approach for each disease is outlined briefly below.

### Direct physiologic effects of heat and cold on cardiovascular mortality

Time-series studies were used to characterize the relationships between temperature variations and cardiovascular disease mortality (global assessment), or all-cause mortality (Oceania), for high-risk populations within broad climate zones (global) and cities (Oceania). Estimates of the mean temperature under each climate scenario were given by “shifting” these distributions according to the projected changes in future mean monthly temperature per spatial unit. The resulting relative risks therefore represent net annual deaths, the balance of increasing risks from high temperatures and decreasing risks from low temperatures. The global study included an adjustment for adaptation to increasing summer temperatures. In both cases the relative risk estimates are used to calculate only attributable deaths but not DALYs because the contribution of a relatively short-term advance of deaths in highly vulnerable individuals to the total duration of life lost is highly uncertain.

### Impacts on diarrheal disease

Dose–response relationships were derived from time-series studies of temperature variations and diarrhea incidence in developing countries (Checkley et al. 2000; Singh et al. 2001). Relative risks were calculated by multiplying the projected increase in temperature by the derived exposure–response relationship. In the global assessment the resulting relative risks were applied to WHO estimates of the overall current burden from diarrhea in developing regions, to estimate attributable diarrhea deaths and DALYs from climate change in 2000 and relative risk estimates for years to 2030 (adjusting for effects of economic development). In the Oceania assessment the relative risks from studies conducted in developing countries were applied to remote Aboriginal populations (which suffer rates of diarrheal disease similar to those of developing countries) to estimate increased diarrhea cases out to 2050.

### Impacts on malnutrition (global assessment only)

Existing crop models were used to estimate the effect of projected changes in temperature, rainfall, and CO_2_ on future yields of grain, cereals, and soybeans (Parry et al. 1999). These crop yield estimates are part of a world food trade model that accounts for the effects of market forces and government policies on prices, trade, and trends in agricultural and technologic conditions. The model estimates the proportion of the population in each region that has access to sufficient food to avoid undernourishment (Food and Agriculture Organisation 1987) within each climate scenario.

### Natural disasters caused by extreme weather and sea-level rise: coastal floods, inland floods, and mudslides

Relative risks of health impacts from rising sea levels were derived from published models that assess the contribution of projected sea-level rise, topography, and population distribution to estimate numbers of people likely to be exposed to flooding in the future (Hoozemans and Hulsburgen 1995; Nicholls et al. 1999). A new model was developed to estimate the effect of increasingly extreme rainfall events on the impact of inland floods and mudslides on human health. This model was based on an *a priori* assumption that inland flood/landslide frequency is proportional to the frequency with which monthly rainfall exceeds the highest value that might occur once every 10 years, under baseline (i.e., 1961–1990) climate conditions. The assessment in Australia was able to take advantage of a high-quality historical rainfall record, which provided a longer time series to estimate the baseline mean and variability distribution than could be collected at the global level. The future change in frequency of such extreme events was mapped against future population estimates to give the per capita change in risk of experiencing such an extreme weather event. The relative risk for each geographic region was applied to the baseline rate of flood death and injury [derived from reports catalogued in the Emergency Events database (EM-DAT)] [Office of U.S. Foreign Disaster Assistance/Center for Research on the Epidemiology of Disasters (OFDA/CRED) 2001]. The models of flood risk from sea-level rise incorporate the adaptive effect of improved flood protection, assumed to correlate with increasing gross domestic project (GDP) over time. In the global analysis an equivalent adjustment was made for the effect that an increasing GDP was assumed to have on protecting against inland floods.

### Vectorborne diseases

Several approaches exist for examining the effect of climate change on vectorborne diseases, based either on observed relationships between climate conditions and vector development in laboratory or field studies (biological or empiric models) or on correlations between the geographic distribution of disease or vectors and climate variables (statistical models). In the global assessment a validated biological model of the influence of climate on the distribution of *falciparum* malaria in Africa (Tanser et al. 2003) was used to estimate the relative change in population exposed to transmission throughout the world under the alternative climate scenarios. In the Oceania assessment, a locally derived biological model was used to estimate future changes in climatic regions suitable for maintenance of the malaria parasite and vectors within Australia (Bryan et al. 1996), and a global statistical model (Hales et al. 2002) was used to predict regions where dengue transmission could occur in Australia and the increase in regions suitable for dengue transmission in Pacific Island nations. The level of spatial detail in the final models enabled a preliminary estimate of the future costs that might be needed to retain a similar level of protection against these diseases in northern Australia.

Estimates obtained using the methods described above are summarized in Table 2 for the Oceania assessment and Table 3 for one region within the global assessment.

## Discussion

There is a general consensus in the scientific literature that human actions are contributing to climate change (IPCC 2001b; Oreskes 2004). Many diseases of public health significance are highly sensitive to climate variability and are likely to be affected by the observed and predicted trend toward warmer and more variable climate conditions (e.g., McMichael and Githeko 2001; McMichael et al. 2003a; National Research Council 2001; Patz et al. 2005). Therefore, to inform mitigation decisions, policy makers are increasingly interested in the likely direction and size of these health effects and their interaction with other nonclimatic influences. To plan adaptation measures, these policy makers also require information on the most important disease threats that climate change may bring to specific populations.

Ideally, policymakers considering a particular decision should have quantitative estimates of the full range of effects on human health, over the duration of effect of the decision (e.g., actions to reduce greenhouse gas emissions should be considered over the entire period for which greenhouse gases persist in the atmosphere and influence climate). The comparative risk assessment provides a framework in which to work toward this aim. It is based on standard epidemiologic methods, that is, the definition of a theoretical minimum exposure to the risk factor, measurement of current and projected future exposure levels, consideration of the strength of evidence of an association between the risk factor and various health states, measurement of the relative risk of suffering the disease under alternative exposures, and adjustment for the effects of confounders or effect modifiers. The methods are therefore transparent and open to challenge and further refinement. For example, recent studies of the effect of climate change on measures of malaria risk at national (Hartman et al. 2002) or regional (Small et al. 2003) levels could potentially be used to reestimate or to provide sensitivity measures of this specific impact. The global assessment shows how summary measures of population health, such as DALYs, further allow the different potential health effects of climate change to be aggregated into a single metric, so that the total (measurable) effect can be compared with that of other health risk factors. The Oceania regional assessment demonstrates how different health impacts can be represented separately, illustrating variation in risk between geographic regions and subpopulations, which may be more transparent and intelligible for policy makers.

Such assessments therefore have important advantages. First, by aiming at a comprehensive assessment, they give a better representation of the health consequences of climate change than studies of single disease outcomes in restricted populations. Second, they help to identify the relative public health burden of different climate-sensitive diseases. The global assessment, for example, showed that relatively small proportional increases in risk for climate-sensitive diseases such as diarrhea and malnutrition may cause very large increases in the total future disease burden.

The attempt to carry out a full accounting of the health impacts of climate change rapidly clarifies significant knowledge gaps. Most of the climate–health models estimate the effects of changing mean values of a climate condition, usually temperature, whereas there is increasing evidence that less predictable changes in extreme values (e.g., Zhou et al. 2004), particularly of precipitation (e.g., Small et al. 2003), may be more important for many diseases. The outputs of many models relevant to such assessments [e.g., predictions of changes in the land area suitable for malaria transmission (Thomas et al. 2004b), population exposed to malaria (Rogers and Randolph 2000), or per capita duration of exposure (Tanser et al. 2003)] are linked only indirectly to disease rates and therefore represent only very approximate measures of the impacts on the burden of clinical disease. Finally, there is still only a limited understanding of the interactions between climate and many important diseases, such as the effects of both temperature and precipitation on diarrhea incidence across different populations. Many plausible or even probable mechanisms by which climate change may affect health have not been modeled quantitatively and have therefore not been included in these assessments. These include, but are not restricted to, changes in outdoor air pollution and aeroallergen (pollen) levels (Beggs 2004), the effect of melting snows and glaciers on floods and landslides, changes in the distribution and transmission of other infectious diseases (particularly vectorborne diseases), the rate of recovery of the ozone hole [affecting exposure to ultraviolet radiation (Shindell et al. 1998)], indirect effects on food production acting through plant pests and diseases, population displacement and destruction of health infrastructure in natural disasters, and the risk of conflict over natural resources.

More generally, quantitative methods generally disregard low-probability but high-impact outcomes. There is increasing evidence from the paleo record and among climatologists (e.g., Hoerling and Kumar 2003) to support a hypothesis that the projected levels of greenhouse gas emissions could lead to a “regime shift” in future climate (driven by, e.g., large releases of methane from the ocean floor, burning and deforestation in the Amazonian rainforest, or a shutdown of the Gulf Stream). Future research in the health area may reasonably assess the health risks—and adaptive requirements—that an abrupt climate change might provoke.

## Challenges to Be Addressed

The particular characteristics of climate change cause an additional range of methodologic issues that may be more difficult to resolve. Compared with more traditional risk factors, actions to mitigate or adapt to climate change affect human health through a much wider variety of mechanisms and over much longer periods. Models vary in the extent to which they account for changes in nonclimatic confounders such as the protective effect of adaptation, socioeconomic development, and technologic advances or, conversely, increased vulnerability through population aging and inequity in income or health care provision. Improved health surveillance data, more detailed epidemiologic analysis, and collaboration with nonhealth disciplines should help to narrow these uncertainties. The comparative risk assessment framework described here attempts to estimate only the consequences of changing levels of the risk factor rather than the total effect of any intervention to reduce the risk factor. Examples include ignoring the health co-benefits of reduced air pollution (Cifuentes et al. 2001a, 2001b) or, conversely, the possibility that interventions that reduce fossil fuel consumption may suppress economic development and therefore health status.

There are several levels of uncertainty inherent in the process of estimating climate change health risks. Because anthropogenic climate change is a long-term phenomenon that is superimposed onto natural climate variability, there will always be some uncertainty around the attribution of health impacts, particularly at the national or regional level. Two major types of uncertainty surround the estimation of the exposure measurement. First, we still have incomplete knowledge about how the climate system will respond to continuing change in the composition of gases in the atmosphere. Second, we cannot know in advance what social, technologic, demographic, and behavioral changes will occur in human societies over coming decades. For these reasons, results should not be reported in a way that suggests a higher probability to the central estimate of a series of scenarios.

## Conclusions

The comparative risk assessment framework is a potentially useful approach to presenting policy-relevant quantitative estimates of the risks that climate change poses to health, at both the global and the national level. In presenting these findings to decision makers, it is important to make clear the limitations of these assessments: quantitative estimates are unavoidably uncertain, changes in nonclimatic factors will influence both the baseline rates of disease and their sensitivity to climate effects, and many of the mechanisms by which climate change may affect health are not currently modeled, likely leading to an underestimation rather than an overestimation of health threats.

Given limited public health resources in many countries for risk assessments, it is important to reduce duplication in this work. Global assessments capture the scale of the future climate-change–related disease burden and highlight regions most at risk for particular health outcomes. This can provide a prompt for specialized regional assessments, which in many regions may give enough accurate information to use at the national level—particularly if countries within the region share similar climatic and economic conditions (e.g., estimated mosquito-borne disease burden in countries with equivalent risks and control programs). National assessments can provide the highest level of spatial resolution and hence the opportunity to quantify variation in risk between administrative divisions. They can also be useful to focus political and community awareness on what is now increasingly recognized as a serious public health issue.

## Figures and Tables

**Table 1:** Health outcomes quantified in the global and Oceania comparative risk assessments.

			Assessment	
Type of outcome	Outcome measure	Health measure	Global	Regional
Direct impact of heat and cold	Cardiovascular disease deaths	Incidence	✓	✓
Foodborne disease	Diarrhea episodes	Incidence	✓	✓
Waterborne disease	Diarrhea episodes	Incidence	✓	
Vectorborne disease	Malaria cases; dengue cases	Incidence	✓	✓
Natural disasters^a^	Fatal unintentional injuries	Incidence	✓	✓
	Population displacement	Prevalence	✓	✓
Risk of malnutrition	Nonavailability of recommended daily calorie intake	Prevalence	✓	

✓ , risk assessment conducted.

a All natural disaster outcomes are separately attributed to coastal floods, or inland floods and landslides.

**Table 2:** Summary of main findings of the Oceania (for 2050) risk assessment.

Exposure	Health impact estimated	Baseline health impact	Future health impact
Temperature extremes (cold and heat)	Attributable mortality in > 65-year-old age group	1,100 deaths per year (across 10 cities); temperate cities have higher rates of heat deaths than tropical cities	Annual mortality range from 1,400 to 2,000, depending on scenario; increase in heat deaths will significantly outweigh decrease in cold deaths
Rainfall (inland)	Annual incidence of deaths and injuries	Average annual death rate in Australia (1970–2001) was 0.41/million (state rates varied from 0.05 to 3.1); the injury rate was 1.9/million (range, 0.1–8.7)	Predicted annual death rate of 0.53–0.61/million (state rates vary from 0.06 to 4.8); the injury rate was 1.99/million (range, 0.22–13.77)
Temperature and rainfall	Population living in a potential malaria transmission zone	Imported cases only	Substantial southeastern expansion of the malaria zone
Vapor pressure	Population living in a potential dengue transmission zone	Dengue not established, but local outbreaks from infected travelers occur in far northeast Australia in most years	Substantial southeastern and westward expansion of the dengue zone
Temperature	Annual incidence of diarrheal disease	Aboriginal people living in remote arid communities have high levels of diarrheal disease	A 10% (5–18%) increase in the annual number of diarrheal hospital admissions among Aboriginal children

**Table 3:** Example of findings of the global (for 2030) risk assessment for one WHO subregion (AfricaE: those sub-Saharan African countries with high child and very high adult mortality).

Exposure	Health impact estimated	Baseline regional situation in 2000	Estimated relative risks attributable to climate change under unmitigated emissions scenario
Rainfall (inland)	Annual incidence of mortality from inland flooding	Average 230 deaths/year reported from 1980 through 1999^a^	1.86 (1–2.44)
Sea-level rise and coastal flooding	Annual incidence of mortality from coastal flooding	No deaths reported in 1980–1999^a^	1.18 (1.09–1.35)
Temperature and rainfall	Annual incidence of *falciparum* malaria	More than 420,000 deaths/year^b^	1.14 (1–1.28)
Temperature and rainfall	Annual incidence of malnutrition	More than 900,000 deaths/year from malnutrition-related conditions^b^	1.02 (1–1.05)
Temperature	Annual incidence of diarrheal disease	More than 430,000 deaths/year^b^	1.08 (0.99–1.06)

The effects of temperature extremes on cardiovascular disease deaths are not presented here because of considerations of short-term mortality displacement (see text).

a Baseline data derived from OFDA/CRED (2001).

b Baseline data derived from WHO (2002).

## Appendix

The IPCC SRES (Nakicenovic et al. 2000) has approved four different storylines that describe plausible relationships between greenhouse-gas-emission driving forces (economic growth, technology, etc.) and the future concentration of gases in atmosphere. These SRES scenarios provide the basis for quantification of future emissions.

The A1 storyline describes a world of very rapid economic growth, a global population that peaks around 2050 and declines thereafter, and the rapid introduction of new and more efficient technologies. Major underlying themes are convergence among regions, capacity building, and increased cultural and social interactions, with a substantial reduction in regional differences in per capita income. Within A1, there are three main subgroups, distinguished by their technologic emphases: fossil fuel intensive (A1FI), predominantly nonfossil energy sources (A1T), and balanced across all energy sources (A1B).

The A2 storyline describes a world of regional self-reliance and preservation of local identities. Fertility patterns across regions converge slowly, which results in increasing global population. Per capita economic growth and technologic change are more fragmented and slower than other storylines.

The B1 storyline describes a convergent world with the same population as in A1 but with rapid changes in economic structures toward a service and information economy, an emphasis on global solutions to economic, social, and environmental sustainability, and the introduction of clean and resource-efficient technologies.

The B2 storyline has an emphasis on local solutions to economic, social, and environmental sustainability. Global population increases at a rate lower than A2, with intermediate economic development and less rapid and more diverse technologic change than in A1 and B1.

In all the scenarios, the effect of specific climate initiatives to reduce emissions [e.g., the Kyoto Protocol to the United Nations Framework Convention on Climate Change (United Nations 1998) is not included.
